# Regionally Varying Habitat Relationships in Lichens: The Concept and Evidence with an Emphasis on North-Temperate Ecosystems

**DOI:** 10.3390/jof9030341

**Published:** 2023-03-10

**Authors:** Asko Lõhmus, Jurga Motiejūnaitė, Piret Lõhmus

**Affiliations:** 1 Institute of Ecology and Earth Sciences, University of Tartu, J. Liivi 2, 50409 Tartu, Estonia; 2 Laboratory of Mycology, Institute of Botany, Nature Research Centre, Žaliųjų Ežerų 49, LT-08406 Vilnius, Lithuania

**Keywords:** adaptation, ecotype, ecophysiology, environmental filtering, habitat selection, limiting factors, macroecology, niche, oceanicity-continentality gradient, spatial ecology

## Abstract

Habitat ecology of lichens (lichen-forming fungi) involves diverse adaptations to stressful environments where lichens use specific habitat conditions. Field observations confirm that such habitat ‘preferences’ can vary significantly across species’ distribution ranges, sometimes revealing abrupt changes over short distances. We critically review and generalize such empirical evidence as broad ecological patterns, link these with the likely physiological mechanisms and evolutionary processes involved, and outline the implications for lichen conservation. Non-replicated correlative studies remain only suggestive because the data are frequently compromised by sampling bias and pervasive random errors; further noise is related to unrecognized cryptic species. Replicated evidence exists for three macroecological patterns: (a) regional limiting factors excluding a species from a part of its microhabitat range in suboptimal areas; (b) microhabitat shifts to buffer regionally adverse macroclimates; (c) substrate suitability changed by the chemical environment, notably air pollution. All these appear to be primarily buffering physiological challenges of the adverse conditions at the macrohabitat scale or, in favorable environments, coping with competition or predation. The roles of plasticity, adaptation, dispersal, and population-level stochasticity remain to be studied. Although lichens can inhabit various novel microhabitats, there is no evidence for a related adaptive change. A precautionary approach to lichen conservation is to maintain long-term structural heterogeneity in lichen habitats, and consider lichen ecotypes as potential evolutionarily significant units and a bet-hedging strategy for addressing the climate change-related challenges to biodiversity.

## 1. Introduction

Field biologists have been long aware that a species can inhabit different habitats across its distribution range, sometimes changing its habitat use abruptly within a short distance. Motivations to explain and account for spatial variation in species-habitat relationships are clearest in applied research and involve a range of subjects—from habitat/niche and distribution modeling of species and assemblages, to using species as indicators of ecosystem functioning or of certain environmental qualities (e.g., [1,2,3,4,5,6]). For example, proper use of plants to *indicate* site conditions requires an understanding of spatial representativity of the background data and, where needed, local calibration of indicator values to address shifts in species responses and sampling bias [7,8,9]. In terms of *managing* for the future, consideration of a hidden tolerance niche (i.e., “almost suitable” habitat) outside species’ current range greatly influences extinction rate estimates and management perspectives [10]. It affects, for example, practical necessities to define ‘critical habitat’ for survival or recovery of officially listed species in many countries (e.g., [11]). Observations on varying habitat use across the range are particularly useful for this task. Finally, basic research on the causal mechanisms of geographic patterns might improve our ability to *predict* species-habitat relationships in space and time [5,12,13,14,15]).

Several ecological features of lichens—stable multi-partner fungal-algal associations controlled by lichen-forming fungi [16]—make them distinct objects for habitat studies from a geographic perspective. First, many lichen-forming species reveal large and unexplained distribution patterns, such as various disjunct ranges [17]. One could thus expect significant geographic variation in lichen environments, favoring a diversity of responses through plasticity and acclimation [18], switching to alternative algal (phototroph) partners [19,20] or adaptive divergence [21,22]. At the same time, a scarcity of taxonomically useful morphological or chemical characters implies that many historically described lichen-forming species involve multiple evolutionary lineages that may be habitat specific and merit recognition as full species (e.g., [23]). Molecular taxonomic revisions are still rebuilding this basic knowledge of lichen distribution and habitat patterns [24,25]. 

Secondly, lichens are fundamentally rather stress-tolerant organisms that are common in extreme environments [26] where they meet distinct limiting factors and physiological challenges. Their central fungal-algal symbiosis itself can be seen as an adaptation against environmental stress [27,28]. In productive environments, lichens encounter diverse interactions with plants and animals [29], which may involve specific limiting factors such as shading by faster-growing vegetation, nutrient excess, or predation [30,31]. 

Thirdly, lichens inhabit a range of distinct substrates—from rocks to plant leaves. This simplifies natural-history comparisons across the globe. The substrate use patterns may also provide evolutionary insights (e.g., [32]), given that discontinuities of habitat types predispose populations to ecological speciation [33]. Disruptive selection may thus be frequent in the populations of lichen-forming fungi. Lichens are also conspicuous colonists of various artificial habitats, notably artificial stony substrates, building timber, artificially exposed ground and rocks, even glass and plastic. Artificial surfaces have been long recognized to host distinct lichen assemblages (e.g., [34]), and old buildings have become vital for some threatened populations [35,36,37]. Globally widespread artificial substrates offer opportunities for standard regional comparisons that may be confounded for natural substrates that vary enormously.

Understanding geographic patterns in lichen ecology is particularly relevant for applied ecological research on bioindicators, habitat conservation, and prediction of future assemblages. For example, the popular concept of “old-growth indicator species” appears to perform differently among regions [38,39]. Yet assigning regional “ecological indicator values” (analogues of Ellenberg’s values) to lichen-forming fungi remains obscure in terms of the spatial representation of the background data [40,41,42,43,44,45]. The situation is similar for other bioindication issues of lichens: geographic variation is considered noise rather than a research issue (e.g., [46,47]); at best, regional indicator species lists have been distinguished (e.g., [48]). The climate change impacts on lichen-forming fungi have been predicted for only a small subset of habitat generalists, without much attention on potentially changing habitat-relationships [19,49]. Stable habitat relationships are also an implicit assumption in habitat conservation, which is the main approach to protecting threatened lichen-forming species [30]. Since habitat management practices usually follow jurisdictions, managers need to know what can be learned from the experience elsewhere.

A modern synthesis on geographic (regional) variation in habitat relationships of lichen-forming fungi is lacking; the evidence remains largely anecdotal and poorly explained (Section 2.2). Several striking examples of regional substrate use can be found in the classic texts by Barkman [50] and Brodo [51], and the latter calls for more covering analyses. Both of these authors mention also geographic variation in substrate specificity, and Brodo [51] mentions local adaptation as a possible cause. In the 1980s, the monographs by Kershaw [18] and Kappen [52] on lichen physiology in relation to their microenvironments treated geographic variation mostly at the interspecific level, but both also highlighted a large phenotypic plasticity involved. Later, the issue has received interest mostly at the level of case studies, being absent from major lichen biology texts (e.g., [27,53]). 

In this paper, we synthesize the evidence on regionally varying habitat relationships in lichen-forming fungi. We first distinguish the main types of such evidence and methodological problems encountered (biased sampling; statistical errors; incomplete taxonomic knowledge). Most field observations originate from north-temperate ecosystems, and we add some original data in critical knowledge gaps. We then organize the evidence by the likely proximate mechanisms and ultimate causes involved: ecophysiological responses of functional thalli, dynamics and demography of populations, and the evolution of intraspecific habitat-specific lineages. Finally, we outline the main conservation implications—for understanding, and responding to, habitat-based threats to lichen diversity.

## 2. Materials and Methods

### 2.1. Key Concepts

We use the habitat concept in its organism-centered meaning: as a space suitable for an organism to use [54]. Habitat is a realization of the (Hutchinsonian) niche, basic requirements of an organism. Hutchinson [55] acknowledged that a multidimensional hypervolume of *fundamental niche* (“the limiting values permitting a species to survive and reproduce”) can be only manifested in those factor combinations that are present in real environments (calling it a “biotop space” at a given moment). We use the term *habitat requirements* for such range of potential habitats. The distinction between *realized niche* and *habitat use* is less clear, because the former is a mixture of the theoretical niche space and actual conditions (notably competition pressure) in Hutchinson’s [55] approach. However, potential habitats may remain unoccupied by an organism for many other reasons, such as limited dispersal, population’s demographic potential to expand or large-scale population dynamics [56]; in lichens, also due to photobiont scarcity for sexually produced fungal spores ([57], but see [58]). For consistency, we refer to any observed habitat occupancy patterns as *habitat use*, which can be characterized in terms of *habitat specificity* (a relatively limited range of habitats used) and *habitat preferences* (disproportionate use of the environment accessible to the organism). Finally, *habitat quality* refers to the capacity of a habitat to provide conditions for individual or population persistence [54]. In lichens, habitat quality can be quantified by combining their vitality, fertility, and abundance measurements ([50], p. 165).

We distinguish two spatial scales of habitat: (i) *microhabitat* (habitat for a thallus or a functional individual *sensu* [30]), such as a host tree or its part, rock surface, patch of ground etc., and (ii) *macrohabitat* (habitat for a population of functional individuals), such as a forest stand, meadow, or water body. The heterogeneous macrohabitat scale, which can host several populations is termed *landscape*. Similar types of microhabitats are referred to as *substrates* (e.g., [51]), and similar macrohabitats as *habitat types*. We call any spatial or temporal intraspecific difference in habitat use as a *habitat shift*; the term *habitat switch* has been reserved to colonization of new habitat types. 

For practical purposes, we do not distinguish habitats based on *microenvironment* characteristics that cannot be measured without special equipment (microclimate, substrate chemistry, etc.). For example, a host tree species or a type of rock is viewed as the “same microhabitat” for a lichen across regions, although there is inevitably variation in its microenvironment conditions. The significance of microenvironment for lichen ecology is nevertheless worth detailed study, since also the opposite can be true. Thus, certain soils and rocks may be structurally different but chemically similar, explaining habitat switches between these two substrates [59]. 

We restrict our treatment of lichens to the ecologically obligate and stable self-supporting association between an ascomycete (or in a few cases a basidiomycete) fungus and algae or cyanobacteria ([60], but see [16] for a discussion). We ignore the rare case of optional lichenization that is the life strategy of some saprotrophic fungi [61]. Although biodiversity and conservation studies usually include with lichens also some other groups of ascomycetes (such as saprotrophic calicioids or lichenicolous fungi), the latter differ from lichens in basic carbon economy and the lack of ‘lichen substances’, which are crucial for lichen habitat relationships. Thus, lichens acquire carbohydrates from the photosynthesis of the photobiont—a process typically limited by environmental light and thallus water content [62], while saprotrophic/lichenicolous fungi derive fixed carbon from plant or lichen tissues [63]. Lichen substances (carbon-based secondary metabolites of the fungus) have numerous specific protective roles, ranging from herbivore and parasite defense, to sun-screening and molecular defense against oxidative stress and toxic compounds [64,65]. We acknowledge, however, that for example, non-lichenized calicioid fungi can share with lichenized species some geographic habitat patterns and conservation issues, such as their dependence on old-growth forests [38]. 

We use a practical concept of *species* as “groups of individuals [of lichen-forming fungi] separated by inheritable discontinuities and which it is useful to give a species name to” [66]. Such definition can also include traditional morphology- and chemistry- based species descriptions until these have been re-examined based on molecular phylogenetic studies. It should be noted that a genetic *individual* cannot be equalized with an individual thallus of a lichen, which may comprise a mixture of genotypes of the lichen-forming fungus [27,67]. For this study, it is not a critical distinction since we assume all genotypes within a thallus or local colony being exposed to the same environment, even though its influence may perhaps differ among genetic mixtures. A species may include *ecotypes*—populations exhibiting habitat-related polymorphism in life history traits. However, in practice, it may not be easy for lichenologists to distinguish habitat generalists, ecologically diverse species, and ‘morphospecies’ that contain mixtures of cryptic lineages. Again, not all of our questions are sensitive to the taxonomy used (e.g., studies on limiting factors in different environments), and circumstantial evidence can help to select among alternative explanations.

### 2.2. The Evidence Considered

**Figure 1:**
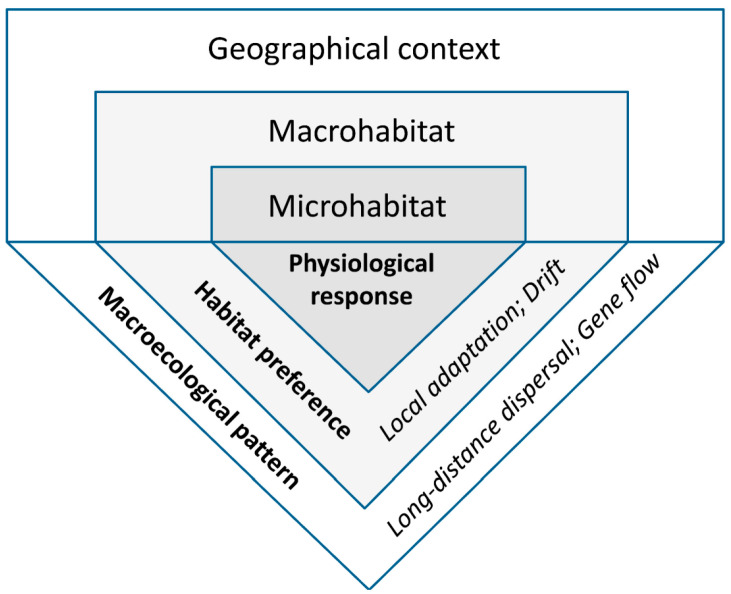
Scale dependence of the main lichen-habitat relationships addressed in this review. The environment affects lichens at three relatively distinct spatial scales (**upper panel**), where their physiological and ecological responses (**lower panel**) can be measured (in **Bold**), while the eco-evolutionary processes can be typically only inferred (in *Italics*).

The environmental conditions experienced by living organisms always vary in space. We focus on repeated macroecological patterns, with proximate and ultimate causes potentially shared among species or regions. To extract, evaluate, and interpret such patterns, we used a framework of hierarchically arranged spatial scales linked by processes (Figure 1), and distinguished three non-exclusive types of evidence that have a biological basis (Section 2.2.1, Section 2.2.2 and Section 2.2.3, respectively). 

In addition to the evidence based on lichen response, we have reviewed local habitat differences of applied value that might imply regional or local adjustment of bioindication or habitat management techniques (e.g., substitute habitats). In such practical cases, variation in habitat relationships can matter even if its causes are unknown or directly anthropogenic. For example, some Central-European remnant populations of the ancient-forest ‘*Lobarion*’ assemblage inhabit rock outcrops, which presumably retained a necessary habitat continuity that was lost in managed forests [68]. Kuusinen [69] attributed a stronger affinity of moisture-demanding lichens to spruce swamps in southern than middle boreal Finland partly to a legacy of former forest-use.

#### 2.2.1. Local Selection Pressures

At the smallest scale, microhabitat affects physiological state of individual thalli. This can be location-specific. Comparative descriptions of selective environments (“lichen environments” *sensu* [18]) can explain local habitat preferences, indicate their development and, perhaps, microevolutionary processes. The latter are more likely for the fungal partner [70] and may also affect generalists via spatially segregated sets of distinct microhabitats (substrates) or by chance (founder populations established by rare long-distance dispersal). For example, natural habitat patches for nitrophilous lichens are highly divergent in terms of the environmental pressures (seashores, riverbanks, treetops etc.; e.g., [71,72]). 

The main problem with this type of evidence in lichens is the measuring of the selection pressure at the individual level. Field studies on *lichen recruitment* are complicated since their progeny cannot be followed, and the contributions of different reproductive modes remain unclear. In fact, reproductive modes may alternate along microhabitat gradients; a study on the epiphytic *Lobaria pulmonaria* reported apothecia to develop more often near tree bases, while isidia were more abundant higher up [73]. In sexual species, the fertile state may (instead of habitat quality) depend on overcrowding, parasite accumulation, or the frequency of mating-type genes (e.g., [74,75,76]). *Growth rate* is routinely reported in ecophysiological studies, but its selection value for lichens varies. Thus, in unstable conditions, rapid growth can enable some long-living macrolichens to attain the size or age required for reproduction (e.g., [77,78]) or for adjusting the water-holding capacity to local water supply [79]. However, as a component of lichen life-history strategies, rapid growth contributes most to overgrowth competition in a particular range of environments unsuitable for plants (e.g., [80,81,82]), since unstable conditions rather favor small-sized taxa and rapid turnover of generations [83]. 

The clearest evidence on disruptive selection pressures on lichens could be inferred from *microhabitat-specific mortality* and related manipulative experiments, such as transplanting to uninhabited sites as practiced in pollution research [46,84]. The value of such experiments is illustrated by the evidence on climatic adaptation in vascular plants; fitness-associated alleles may be neutral in their typical climatic range, but deleterious outside [85]. The experimental techniques may require improvement, however, since typical lichen transplants and their competition-free attachment sites may miss some key factors of the natural habitat [86]. 

So far, research on local habitat-specific mortality of lichen thalli is surprisingly rare, and we are not aware of such studies across regions. Indirect evidence comes from some studies where lichen mortality is related to ecological factors that might produce broad-scale variation in the selection pressures. Thus, local predation by snails on *Lobaria* species can limit their populations (notably juveniles) in calcareous forests, in shady microhabitats, and on particular tree species [87,88,89,90]. A study on colony extirpation in the epixylic *Xylographa parallela* concluded that the colonies in shaded sites are more vulnerable to advanced wood decay [91]. The aspen-specific epiphyte *Ramalina sinensis* appeared confined to very young stands, possibly because the thalli become heavily parasitized in old forests [92]. Such mortality agents are likely to vary in space and thus suggest potential mechanisms for context-dependent microhabitat use [93]. However, their links to local adaptation are less clear and should also consider variation in the mortality patterns. For example, predation can be highly stochastic already at small scales or among years [88,94], which is more likely to select for phenotypic plasticity or, perhaps, polymorphism (diversified bet hedging) in habitat use and herbivore defense mechanisms (Section 4).

#### 2.2.2. Among-Population Variation in Habitat Preference

In lichens, habitat preference studies are restricted to inferences from population patterns because individual spatial choices (‘behavior’; typical of animal studies) cannot be tracked beyond thallus growth. The studies can address (i) context-dependence of habitat use, derived from multifactor analyses of field data, or (ii) causes of distinct habitat preferences. For the latter to cover adaptive, inheritable mechanisms, common-garden experiments should be added to in situ observations (Section 4.2). 

Field evidence of type (i) provides most of the material relevant to our review (examples in Table 1). The most convincing studies have measured habitat availability through balanced representative sampling and/or habitat modeling, but most reports remain anecdotal, qualitative, and/or presented as discussions of case studies. For example, Brodo [51] already thoroughly discussed the occasional occurrence of lichens on non-characteristic substrates and shifts of saxicolous lichens to bark and wood; yet only recently have some of such observations been structured to reveal geographic patterns. Collectively, the evidence confirms, however, that habitat preferences vary across the range in many species of lichen-forming fungi, in various ecosystems and at different scales, and this phenomenon is caused by multiple mechanisms. 

#### 2.2.3. Parallel Shifts in Life-History Traits and Local Habitat Use

Regionally distinct or habitat-type specific life-histories suggest plasticity (including its evolution) or adaptation and genetic drift in the conditions of restricted gene flow. For example, a tendency that vagrant (unattached) macrolichens inhabit dry steppe habitats appears both at the interspecific scale, as well as in the optional vagrancy of some species [110]. In the latter, vagrant phenotypes can form a non-random subset of attached (“normal”) phenotypes and thus suggest an evolutionary adaptation process [111]. Conclusive evidence on adaptation should, however, include evidence on the selection pressure. The latter alone does not confirm a particular evolutionary outcome (Section 2.2.1), while habitat relevance alone cannot confirm the character evolution due to habitat-related pressures. Overall, character evolution remains poorly understood in lichens and often involves parallel appearance and convergence in distant lineages (e.g., [112]). As a result, intraspecific geographic variation in key traits, through revealed in a number of lichen studies, is difficult to relate to specific habitat-related processes (Section 4).

Perhaps the best examples of this type of evidence involve regional modes of reproduction—reflecting environmental pressures on the lichen to re-allocate its resources and/or on its ability to colonize new substrates or sites. Based on an interspecific comparison of parmelioid lichens, Lawrey [113] concluded that mixing reproductive modes provides stronger selective advantages in temperate than in tropical areas. He attributed this to a higher variability and unpredictability of temperate environments (cf. [114] for a criticism of this idea based on animal studies). However, in heterothallic species (such as *L. pulmonaria*), unstable conditions may also accelerate genetic drift or induce population bottlenecks, suppressing sexual reproduction through an unbalanced ratio of mating-type genes [76]. In fact, any process reducing reproduction can constrain “resource-tracking” abilities of poorly dispersing lichens, such as *Usnea longissima* in the Pacific Northwest [115]. In contrast, optimal climatic conditions may accelerate lichen growth to reproductive states so much that new macrohabitats containing only short-living substrates become available [78]. 

One of the most comprehensive studies is by Lidén and Hilmo [116] on the hydrophilic macrolichen *Platismatia norvegica* in Scandinavia. In terms of habitat use (Section 2.2.2), they showed that *P. norvegica* is restricted to riverine sites in the suboceanic region, and its tree-scale abundance increased with the proximity to stream and with bark pH. Such preferences were absent in the oceanic sites, indicating wider habitat use (tolerance) and different limiting factors. In terms of life-history traits, *P. norvegica* thalli in the suboceanic region were smaller and more densely covered by diaspores; this suggested either slower growth or allocation of more resources to reproduction. The suboceanic thalli were also less parasitized. The practical conclusion was that riverine sites in the suboceanic areas can effectively act as refuges for hydrophilic lichens of conservation concern.

### 2.3. Methodological and Interpretational Problems

Because of the predominance of observational and correlational approaches, broad-scale habitat studies are prone to bias and misinterpretation. For example, a lack of consistency among locally derived lists of putative old-forest-dependent species does not support a geographic pattern, but rather reveals a mixture of unaccounted local variation and methodological issues [38]. We distinguish three major pitfalls: (i) regionally biased sampling; (ii) random error accumulation due to multiple testing in assemblage studies or in multifactor habitat modeling; and (iii) mixtures of cryptic species instead of an ecologically polymorphic single species. 

**Figure 2:**
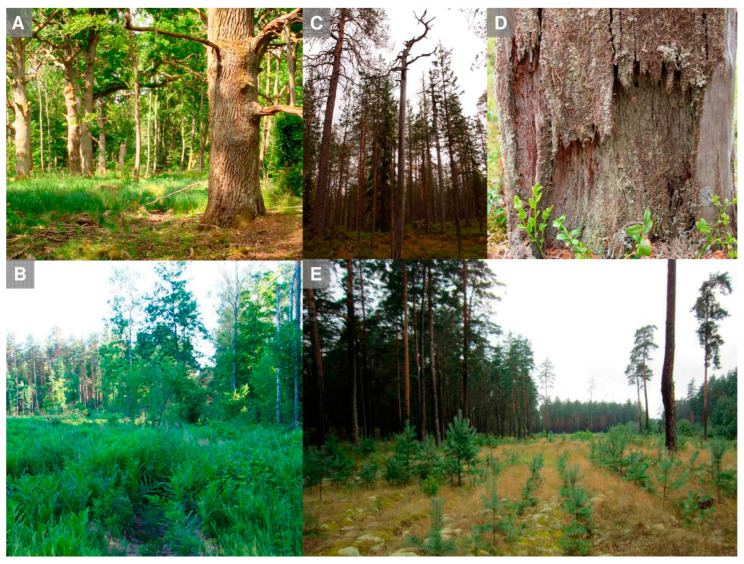
Variation of typical macro- and microhabitats across the European range in *Cladonia parasitica*, a specialized wood-inhabiting lichen. (**A**) Pedunculate oak (*Quercus robur*) stand in South Sweden, where it grows on dead branches, trunks, and stumps. (**B**) Clear-cut of a Scots pine (*Pinus sylvestris*) dominated stand in SE Belarus, where found on an oak stump. (**C**,**D**) Primeval boreal forest in Finnish Karelia, on old wood of decorticate Scots pine snag (‘kelo’). (**E**) Stumps and logs in managed pine forests in Lithuania. A and C illustrate well-known, regionally distinct habitats [107], while B and E represent historically poorly studied habitats (habitat bias). Photo courtesy: U. Arup (**A**), P. Lõhmus (**B**,**E**), A. Lõhmus (**C**,**D**).

A major sampling issue is how to compare populations based on regionally inconsistent habitat sampling. *Habitat bias* may be overwhelming in heterogeneous datasets, which pool multiple casual surveys, such as museum collections or floristic databases (e.g., [39,117]). For example, *Cladonia parasitica* was considered a typical dead-wood dweller of old forest (thus of conservation concern in Northern Europe; see also Figure 2), until targeted sampling found it to be frequent on clearcuts in dry pine-dominated sites [118]. Subsequent standardized fieldwork in Finland, Lithuania, and Belarus confirmed that this is not a local habitat use pattern (P. Lõhmus, unpubl. data). In epiphytes, the usually poor sampling beyond human vertical reach [119] is a potential source of regionally incomplete habitat descriptions. *Sufficient sample size* is particularly important for analyzing habitat specificity (niche breadth). Thus, in their comprehensive study on wood-inhabiting lichens, Spribille et al. [98] defined “obligate lignicoles” by >99% of occurrences on wood. Such a criterion is, however, very data-demanding for a robust analysis since, for each species, one would need hundreds of records from similar sampling approaches in the regions compared.

Especially with small samples, a bias may arise due to a *lack of independence* among field records or herbarium collections. Specifically, clonal thalli formed in vegetative reproduction can be considered as “parts of one fragmented individual” [120], but this cannot be distinguished in the field. If there is a genotypic predisposition for habitat use, extensive sampling in a small area could introduce pseudoreplication [121]. Some common-sense weighting of closely distributed records might help: for example, counting thalli on one tree or on a limited ground area as one “functional” individual [30,122], and dividing those records between the different microhabitats observed [38]. Whether such procedures actually increase the rigor of lichen habitat studies has not been assessed. 

Multifactor habitat modeling or species-level interpretation of assemblage data (e.g., indicator species analyses) are well-established tools of lichen habitat studies. However, because these procedures usually include multiple tests, they are prone to inference derived from exceptional observations (Type I errors). A unique analysis by Will-Wolf et al. [123] on forest lichen assemblages over three spatial scales (two local plus a regional scale) serves as an example. First, after half of all species (found in 1–2 plots) were excluded, 28 species of the 181 remaining were found to contribute to assemblage ordination axes (i.e., belonged to certain assemblages) at both local scales. Of these, only 11 species showed a consistent response to habitat characteristics (such as temperature, air quality, or vegetation type), with a single species (*Parmelia sulcata*) performing similarly in both regions. The authors concluded that “most lichen species are likely to be useful indicators of ecological conditions only within narrow environmental contexts and scale ranges”. That may be true, but requires replicative study given the share of statistically ‘significant’ cases around (the 11 species) or well below (the single species) the commonly accepted 5% risk of random error. Briefly, caution is needed when attributing geographic explanations to differences between studies and datasets [38].

For our treatment, two situations of the *cryptic-species problem* occur. First, when a described species includes closely related allopatric and habitat-specific lineages, which may deserve species status (e.g., [124]). Such mixtures do not necessarily undermine (at least ecological and physiological) inferences to regional habitat relationships, particularly if the speciation in this group has involved habitat-related pressures. A more misleading situation occurs when the taxonomic mixture within a described “generalist species” includes both specialists and generalists in partial sympatry, especially when these are polymorphic and belong to distant lineages (cf. [24]). For such ‘collective species’, various artifactual habitat-use patterns in the geographic space may emerge.

These pitfalls collectively suggest that the ecological studies on geographic variation should become more rigorous. Assemblage-scale data collection can serve as a cost-effective screening phase across multiple taxa and environmental gradients, but it should be followed by in-depth studies on selected species with established phylogenies and based on multiple types of evidence. Historical reports on habitat patterns in the lichen groups with debatable phenotypical boundaries between species are only suggestive without such insights [125,126]. 

## 3. Causal Mechanisms: Ecophysiology and Demographic Processes

Mechanistic explanations to any patterns in lichen-habitat relationships include the responses of individual thalli or propagules to their microenvironment (microclimate, chemical, and structural properties), to the pressures of competing plants, predators and parasites, and to stochastic events. In general, lichen thalli are highly exposed to adverse conditions: their water content depends on the environmental moisture, the exchange of gases and soluble substances proceeds from the whole surface, and—in the absence of roots—lichens’ crucial but unregulated ability to concentrate nutrients from the atmosphere exposes them to contamination [27]. A high exposure implies that individuals closely respond to their environment throughout a species’ distribution range either by phenotypic plasticity, acclimation and recovery mechanisms, and/or colonization of favorable habitats. Some lichens have an enormous potential of such responses. For example, simulation chamber experiments demonstrate that the psychrophilic crust *Pleopsidium chlorophanum* would successfully acclimatize in the almost oxygen-free conditions of the planet Mars if it could inhabit rock fissures for being protected from the lethal irradiance [127]. Even the driest deserts can have lichen microassemblages attached to small particles and being able to rapidly respond photosynthetically to fog events [128]. Given such ‘extremotolerance’ plus abilities to colonize buffering microsites, it is not obvious why, when, and how lichen-forming species should shift their habitats.

**Figure 3:**
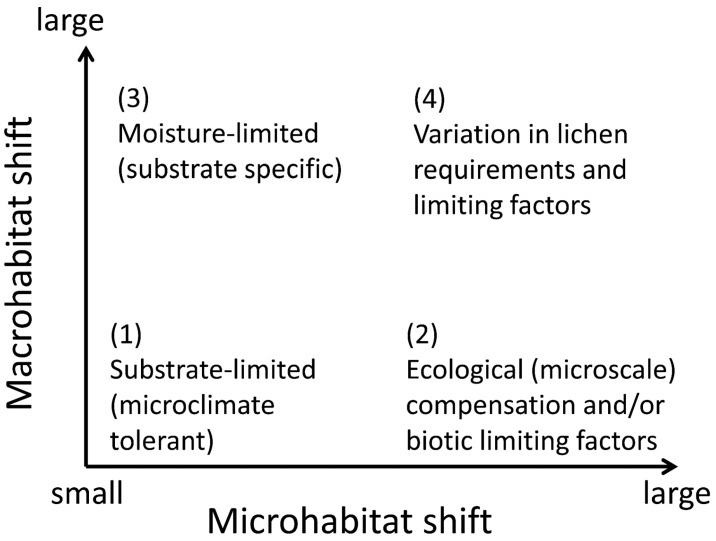
A framework of habitat-use responses to ecophysiological challenges posed by large distribution ranges in lichens. The responses include shifting microhabitats within a macrohabitat, consistent microhabitat use across varying macrohabitats, and shifts at both scales or none at all.

We review two basic sets of mechanisms that can create geographic patterns in lichen-habitat relationships. The *ecophysiological mechanisms* are apparently more common; these combine environmental filtering and biotic exclusion at the thallus or propagule scale. We distinguish four patterns that differ in their relative shifts in micro- versus macrohabitat, depending on the ecophysiological background: (1) wide microclimate tolerance; (2) broad-scale modifiers of microhabitat suitability; (3) habitat release in a favorable macroclimate; and (4) spatial patterns of limiting factors (Figure 3). Our framework is based on research along climatic and edaphic gradients, but the principle can be extended to anthropogenic gradients of land-use and pollution. We acknowledge several difficulties with operationalizing this approach: it is difficult to formalize habitat shift measurements across scales; the shifts in nature are probably gradual, dynamic, and mixed in different parts of a species’ range; and habitat “similarity” is conceptually vague given the different perspectives of a human observer and a lichen ([18]; see also Section 2.1). We, therefore, encourage theoretical research to improve the process-based conceptualization.

Another set of mechanisms is related to *demographic processes*: dispersal, fluctuations in population structure, and stochastic events can affect regional habitat-use patterns within lichens’ tolerance range. These processes can also create local patterns of disproportionate use of the lichen environment, but their persistence (e.g., absence in certain quality habitats caused by chance events) is not known. Furthermore, a wider habitat range observed (e.g., more phorophyte species occupied; Table 1) may partly result from local population size (for any reason) when habitats differ in their quality. Investigation of such patterns is expectably difficult in the case of delayed, hidden, and cumulative impacts of past events, such as recovery from disturbance, or a history of repeated exposure to contaminants [129,130]. An important study on epilithic lichens found that their colonization rate of habitat patches is related to both the local abundance and range size [131]. This could imply that stochastic mechanisms behind regional peculiarities in habitat use could be rather expected in poor dispersers with moderate range sizes.

Substrate-specific species can shift to particular habitat types (Figure 3: response (3)) simply because their substrates are regionally available there. For example, forests may host saxicolous lichens depending on the presence of rock surfaces; open landscapes may host corticolous or epixylic species depending on the presence of single trees. Such cases may require some physiological plasticity (cf. Section 3.1), but otherwise are outside our scope (but note their significance for regionally adjusted habitat management; Section 2.2).

### 3.1. Microclimate Tolerance Based Responses

Using structurally similar habitats across an extensive or heterogeneous range (Figure 3: response (1)) is an informative ‘null model’ of geographic habitat variation. As a concept, it implies that the micro-environmental variation experienced by a species follows that of the macroclimate. Balancing the physiological challenges posed by such micro-environmental variation would require considerable physiological tolerance (acclimation), adaptations, or plasticity. These phenomena are well documented in habitat generalist lichens (e.g., [132,133,134]), where the accompanying habitat variation can, however, buffer some micro-environmental challenges (Section 3.2). 

A pronounced acclimation capacity might thus be expected among those lichens that are restricted to special, but widespread substrates—for example, stress-tolerant crusts obligately inhabiting certain rocks, charcoal, resin, or weathered wood of forest trees. We are not aware of broad-scale ecophysiological studies on such species. Instead, a common knowledge is that many lichens with intercontinentally disjunct ranges inhabit ecologically similar conditions [135]. Thus, a pure microclimatic tolerance without any habitat adjustment across the range may be rare in lichens. Nevertheless, several physiological options of such tolerance have been documented. For example, a similar cortical protection ability has been found in two macrolichens collected on sun-exposed rocks and soils in regions with 3–5 times difference in UV-B irradiance [136]. In two other open-habitat species, regionally distinct acquisition of algal cells (chlorophyll content) by the mycobiont has been reported, which affects production-related characteristics of the lichen [137,138].

### 3.2. Microhabitat Shifts for Ecological Reasons

**Figure 4:**
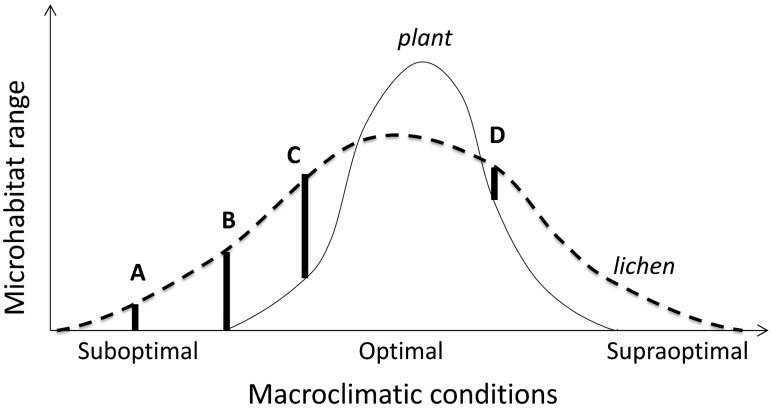
Conceptual model of microhabitat variation along a macroclimatic gradient in a stress tolerant organism (lichen). Its microhabitat range A is constrained abiotically compared to B (environmental filtering *sensu* [142]); while the range C reflects competitive release compared with D. In the optimal climate, it is excluded from all potential microhabitats by a superior competitor (plant). Note that A and D have similar microhabitat niche breadths but different positions (i.e., different microhabitat sets) and their limiting factors. The principle extends to various shapes of the curves provided their partial overlap and consistently asymmetric competition, and to macrohabitat scales where suitable microhabitats are present.

Microhabitat shifts within a habitat type (Figure 3: response (2)) indicate changes in microsite quality due to broader-scale ecological factors, such as regional macroclimate or pollution, or local competition by plants or predation. Such responses can combine, expand to the macrohabitat scale (particularly in response to climate), and form complex species-specific patterns of habitat use (niche realization; Figure 4). 

For example, the cyanolichen *Nephroma occultum* uses a widest range of forest successional stages and microhabitats in macroclimatically suboptimal near-oceanic areas [105]. Tropical epiphyllous lichens may not be able to benefit from regionally abundant rainfall when it simultaneously favors competitively dominant liverworts [139]. Similarly, in British Columbian ‘supraoptimal’ conditions, *Bryoria fremontii* appears to be limited by prolonged wetting, while its habitat use is positively moisture-dependent in its southern ‘suboptimal’ range in California [140]. In wet boreal forests, excess precipitation may limit epiphytes on tree branches also through mechanical destruction by heavy snowpack [141]. 

#### 3.2.1. Climatic Gradients

Shifting of microsites without biotic pressures involved appears to be restricted to microclimate-sensitive species, or to populations at their climatically determined range edges (cf. microhabitat ranges A and B on Figure 4), or in regions where contrasts between habitat types are sharp. For example, snow and ice often limit lichen microhabitat use in cold climates, where particularly severe impacts may occur in open landscapes due to snow drift [143]. In subarctic birch forests, the shade intolerant, but cold resistant, *Melanohalea olivacea* only occupies trunks above snow surface that varies regionally [143,144]. Less sensitive foliose species (such as *Hypogymnia physodes* and *Parmelia sulcata*) show similar patterns in continental areas only [144]. In contrast, the cold-sensitive and competitively inferior *Parmeliopsis ambigua* prefers tree bases and can be snow-covered much of the cold season; this ability is supported by its higher concentration of storage lipids [145]. 

The impact of micro-environmental fluctuations (microclimatic extremes) on lichen habitat use varies along with species-specific protection mechanisms and speed in recovering vital functions, such as cell turgor [146], photosystem II activity [147,148], and (in cyanolichens) nitrogen fixation [27]. For example, since boreal beard lichens (*Usnea* spp.) are susceptible to light during desiccation, they grow higher in the canopies in oceanic regions [149]. Similarly, in the Mediterranean, *Flavoparmelia caperata* appear to be limited to tree bases in drier climates, while it can grow on trunks and even branches in submediterranean climate [150]. For forest epiphytes, the moisture-dependent photoprotection is also involved in edge avoidance: it is clearest in continental climate where drought is common, and in the species that lack photoprotective pigments [151]; see also [152] for an observation of a latitudinal gradient. 

The expectation that distinct microhabitats could substantially buffer environmental adversity has recently received increasing attention due to its relevance for climate change mitigation for biodiversity (*microrefugia*; e.g., [153,154]). For several lichen-forming species and assemblages, a potential to shift their microhabitats has been demonstrated in some ecosystems [155,156,157], but not everywhere [158]. Local opportunities for such shifts can thus be seen as an aspect of long-term habitat quality [19]. For example, exceptional occurrence of several lichen-forming species at 84° S in Queen Maud Mountains, Antarctica, has been attributed to locally steep rock ridges in windy conditions that have provided a persistent refuge against extended duration of snow cover [159]. 

Despite sound expectations, actual climatically induced shifts to new microhabitats across lichen ranges (i.e., beyond degradation of current microhabitats) remain poorly documented. Rather, a recent experiment with *Nephroma arcticum* in Sweden indicated that range edges that follow climatic gradients are not necessarily directly determined by climate [94]. A similarly cautious result was obtained in a landscape-scale study in British Columbia, where the altitudinal ranges >1000 m appeared climatically different, but limited lichen growth similarly because of comparable temperatures during hydration events [160]. The latter indicates the photosynthetically active period as a critical lichen microhabitat property, which is determined by combinations of light (photosynthetically active radiation), moisture, and temperature regimes ([18], p. 40). In conclusion, collecting evidence on climate-buffering microhabitat shifts in different lichen populations remains a topical issue of conservation research.

#### 3.2.2. Environmental Chemical Gradients

Lichen microhabitat shifts can also be due to broad-scale variations in substrate chemistry. For epiphytes, suitability of tree bark is modified by the chemical environment experienced by the tree, including precipitation and uptake from the soil, as well as tree-species specific buffering capacities (e.g., [161,162,163]). Such effects can be also strong on spore colonization, not visible to the human eye [164]. Its regional variations include, for example, marine sediments and natural “sea spray” in oceanic areas that reduce the acidity of tree bark [50,161,165]. This mechanism might partly explain why bark acidity no longer limits *Platismatia norvegica* incidence on trees in an oceanic area [116]. In humid mixed stands in North America, the leachates of canopy trees can locally improve the suitability of surrounding trees (a ‘dripzone effect’; [166,167]). A likely mechanism is downregulating the uptake of some micronutrients (notably Mn and Fe), which achieve toxic concentrations for many lichens on acidic substrates, particularly in the regions with wet winters [168]. At least aspen leachates can be also used by the fungal partner as a carbon source and may thus directly enhance its performance by switching to an alternative nutritional strategy [169]. 

Regional anthropogenic changes in chemical environments (pollution) thus have apparent potential to change lichen habitat distributions. At the microhabitat scale, some shifts are well documented. For example, the historical acidic precipitation in industrial regions caused some corticolous lichens to switch from conifers (naturally acidic) to deciduous trees [170,171,172,173]. Such acidity-driven patterns persist locally until today [174]. Goward and Arsenault [95] even hypothesize that the general scarcity of cyanolichens on conifers in Europe (compared with North America) is caused by the pervasive industrial pollution that has chemically degraded European pristine conifer habitats. 

Some other kinds of pollution (dust; lime; eutrophication) can also blur lichens’ host tree use patterns, but these tend—often in concert, combined with acidity, and strengthened by sun exposure—to homogenize different tree species and microhabitats, which then become inhabitable mostly for a relatively small set of pollution-tolerant lichens [175,176,177]. The ecological effects of nitrogen deposition appear particularly ubiquitous across multiple spatial scales [178], but some microhabitats—e.g., cavities, furrows, and dark forest interiors inhabited by shade-tolerant species—may impoverish more than others [179]. At least alkaline and dust pollution tend to affect the microchemistry and lichens increasingly towards tree tops [180]; thus, one could expect vertical shifts of sensitive species in response to local pollution levels. At extreme levels, pollution can also induce regional habitat switches between corticolous and saxicolous substrates (Table 1; [181]).

#### 3.2.3. Biological Interaction Gradients

Observations of restricted microhabitat use in macroclimatically suitable areas indicate biotic limitation. In lichens, competitive exclusion from certain microhabitats is generally well described, while the roles of predators and parasites and facilitative mechanisms are not (e.g., [182]). Specifically, we lack quantitative estimates of the severity of biotic limitation under changing macrohabitat conditions due to succession, anthropogenic degradation, and climate change (but see [183,184,185]). Those rates might vary, or even alternative pathways arise, for example, due to environmentally induced chemical defense or microbiome, environmental fluctuations, or stochastic limitation of the antagonists (e.g., [186,187]). Most interesting would be the cases when species are biotically excluded from whole ecosystems (macrohabitats) that contain suitable microhabitats.

Lichens represent a model case for such competitive exclusions (conceptualized on Figure 4) because of their sharply asymmetric competition (inferiority) with vascular plants and bryophytes [188], and between slow-growing crustose and fast-growing foliose lichens [82]. The competitive exclusion by plants is clearest in the ground cover, where lichens abound in only those biomes and vegetation types that are too dry, cold or infertile for plants (e.g., [52,188]). The substrate-scale interactions with bryophytes are more complex. In epiphytes, these change from a strong suppression in wet temperate forests [189] to microhabitat enhancement in dry regions where some lichen-forming species may become restricted to moist bryophyte mats [151,190]. Between those extremes, in moist temperate climate, the bryophyte suppression varies among tree and lichen species, and thus structures lichen assemblages [191]. In such cases, the observational patterns should be complemented by experiments [82]. For example, it is unclear whether a reduced microhabitat use and species richness of cyanolichens in the most oceanic sites in the Pacific Northwest is driven by bryophyte competition or heavy snowpacks [95,105]. On Figure 4, these options are depicted as shifts in climatically optimal (C to D) and supraoptimal sections, respectively. 

Although explicit studies are missing, the geographic patterns of deadwood use by lichens (Table 1) are probably related to abiotic and biotic pressures combined (Figure 4, left side). In dry climate, the lack of moisture can restrict lichen use of dead wood [192], while in the case of abundant rainfall and a mild climate, such microhabitats (also tree bases) become unavailable because of enhanced conditions for bryophytes, algae, and vascular plants [193]. Between those extremes, a release from bryophyte competition could partly explain why several lichens are lignicolous in the temperate zone but only facultatively deadwood-inhabiting in the boreal zone (Table 1). However, fallen trees may become less inhabitable also in moist boreal areas due to rapid overgrowth by ground-living bryophytes [194]. 

Compared with plant-lichen interaction, competition among lichens for substrate space is less frequent and primarily related to ‘pre-emptive’ colonization and growth rates [82,183]. Thus, regional factors that facilitate the growth of better competitors (such as some wide-lobed foliose species) have a potential to constrain the regional microhabitat of other lichens. For example, decreases in generalist green-algal lichens on aspen (*Populus tremula*) trunks along the oceanicity gradient in Scotland have been attributed to an increasing abundance of foliose cyanolichens [195]. 

Moderate nutrient enrichment can affect the competitive relationships through differential growth rates [82]. The growth-rate responses depend on species-specific abilities to assimilate nutrients (e.g., increasing photobiont abundance and tolerating higher light levels for their functioning) and to tolerate increased parasitism and plant competition [31,196]. While such general mechanism is evident globally along natural gradients [197], the tolerance of excessive nitrogen pollution is restricted to relatively few ‘nitrophilous’ species that seem to have pathways to rapidly metabolize ammonium to a less toxic storage form [27]. Thus, similarly to the climatic effects, habitat shifts due to regional nutrient enrichment can be competition-mediated in optimal conditions and abiotically limited in supraoptimal conditions (Figure 4). Since the response is dependent on the levels of photosynthetically active radiation, one might also expect increasing avoidance of forests and other shaded habitat types (Figure 3: response (4)), but this has not been studied.

Predation (lichenovory) pressure on lichen microhabitat use has been best documented with regard to snail impacts (see also Section 2.2.1). Snails seem to affect, for example, vertical distribution of some lichens on seashores [198]. In Scandinavia, snails have a role in regional extinction of the threatened *Pseudocyphellaria crocata* populations on rocks and deciduous forests, while this lichen appears safer in sites where it can occupy thin pendant branches of spruce [199]. Interestingly, there is also evidence for indirect impacts mediated by snails in similar (rocky and forested) habitat types. Thus, on limestone pavements in Swedish alvars, snails indirectly enhanced endolithic lichens by consuming a shading cover of cyanobacteria [200], while in forests, wood ants *Formica* spp. can locally reduce snail predation on lichens [201]. These studies reveal that multiple biological interactions can shape lichen habitat use—an issue on which little is known so far.

### 3.3. Broader Range of Habitat Types in Favorable Macroclimates

Regional shifts in habitat-type occupancy for reasons other than substrate availability fall into two categories, depending on whether they also include a shift in substrates. In lichens, repeated evidence for a shift without substantial substrate change (Figure 3: response (3)) exists for one major pattern: increased tolerance of open or disturbed habitats by forest lichens along the continentality-oceanicity gradient. 

A classic example in Europe is the expansion of old-forest lichens of the ‘Lobarion’ assemblage to single trees, tree canopies, and trunks lacking water-holding moss carpets in oceanic climates ([50], p. 523). This may include a broader range of host trees, since faster growth rates enable the lichens to grow also on shorter-living host trees [78]. Later studies have revealed that the set of species involved is much wider [202], and a similar pattern (species increasingly confined to old-growth forests toward continental areas) can also be found in North America [104]. An interesting parallel is the evidence for accelerated evolutionary rates in oceanic areas in collematoid and parmelioid lichens [203,204]. It suggests that a wider habitat range in such areas may be also supported by some regional adaptation (e.g., due to larger populations or mutation rates), either in terms of plasticity or specialization (see also [205]).

An apparent key mechanism behind a wider habitat range along the oceanicity gradient is a sufficient thallus water content to sustain multiple physiological processes: photosynthesis, nitrogen fixation, heat tolerance [27], and photoprotection against excess light [206]. The regional patterns thus differ among species depending on their specific response curves to environmental moisture. For example, the species of epiphytic macrolichens, which are “essentially confined” to old-growth forests in inland British Columbia, are almost exclusively ‘hygrophytic’, and frequently ‘cyanophilic’ [104]. While cyanolichens require relatively high thallus water content for photosynthesis and can only use liquid water, their inhabiting of open habitats is supported by a more efficient water-holding capacity [207] and better irradiation tolerance of the photobionts [151]. 

It is likely that such moisture-driven mechanisms also occur in open ecosystems. For example, in some arid regions the macrohabitat distribution of saxicolous lichens reflects dew formation [208], while that may not be true for other regions with a different main water source. In boreal regions, a general northward tendency of peatland vegetation to expand to mineral-soil areas is acknowledged [209], although this has not been explicitly shown for ground-dwelling lichens thus far. 

The question of how other factors modify the oceanicity patterns of lichen habitat use has not received any systematic treatment. As a synergistic effect, certain marine sediments in the soil can modify tree-bark chemistry in boreal rainforest regions to the extent that the ‘Lobarion’ assemblage successfully colonizes spruce plantations [210]. In contrast, Merinero et al. [211] described habitat patterns in *Lobaria scrobiculata* in the Mediterranean region, which indicated that micro- and mesoscale factors neutralized the oceanicity impacts described elsewhere. Even more extreme examples are from some oceanic urban areas, where meso-scale dryness combined with air pollution effectively excludes many lichens that are abundant in the surrounding landscape [212,213]. Another study system of high conservation relevance is extensive artificially drained wetland landscapes, where one could expect significant habitat shifts in the most moisture-demanding lichens [214].

### 3.4. Spatial Patterns in Limiting Factors

Some parallel shifts in both macro- and microhabitats over extensive ranges of lichens can be expected. Relevant for this review are those shifts that can be predicted, i.e., that form consistent patterns (Figure 3: response (4)). Rather than collecting observations for inductive inference, we here outline some theoretical expectations of how such patterns could be formed. Arguably, the best starting point for their investigation is in macroclimatically adverse regions for whole lichen assemblages, i.e., at joint range edges.

First, we expect patterns that are based on physiological trade-offs. Such basic trade-offs can define alternative micro- and macrohabitat combinations that could sustain a lichen in a particular region. For example, light commonly limits forest epiphytes at tree bases, while water becomes critical higher in the canopy [215]; physiological trade-offs caused by these factors, especially during critical periods, can explain why certain lichens are restricted to particular kinds of forests (e.g., [216]). Similarly, if the defense of photosystems against excessive light is costly (e.g., the production of melanins), it may not support lichen viability in certain ecosystems and regions [217,218]. 

Secondly, photobiont availability might limit lichen populations in particular habitats in a regionally distinct way. In rare cases, fungi can lichenize depending on substrate-specific algal presence, such as in some species of Stictidaceae that become lichenized on tree bark but remain saprotrophic on wood [61]. What appears more frequent is that symbiotic algal genotypes are segregated among habitat types or areas from microhabitat to regional scale, causing geographic variation in symbiosis (e.g., [219,220,221,222]; but see also [223,224]). Consequently, it has been suggested that algal preferences for certain habitats may “lead to the existence of specific lichen guilds” [225]. If that might cause a particular fungal species to be a member of different guilds in different sites, then specific selection pressures could be induced, and regional habitat-specificity might be further favored. The conditions for that are not understood (reviewed by [226]), but the general issue of context-dependent symbiosis resembles that reported for plant mycorrhizal relationships, i.e., it is probably species specific (e.g., [227]).

### 3.5. Demographic Processes

Several population processes could create or maintain regionally distinct habitat use: varying demographic rates, local population events, and expansions to novel habitats. For example, it might be of both evolutionary and conservation interest to explore, which habitat-use peculiarities in small isolated populations result from past population bottlenecks or founder effects (see Section 2.2). Or how the role of dispersal limitation (including limitation at the establishment phase) in distribution patterns is shaped over time through regional habitat configuration and selection for particular reproductive modes (e.g., [228,229]). 

Overall, there is very little evidence on how demographic processes shape habitat use in lichens; these may be indeed most frequent in small populations that lack long-distance dispersal (e.g., [115]; cf. [230,231]). For example, in the regions where substrates are scarce, a dispersal-limited lichen may become confined to the substrate type that is spatially aggregated [232]. Another relevant observation is that burned heathlands were colonized by lichens more quickly in southern than northern Norway [233]. This suggests that the rate of occasional habitat shifts and, consequently, the use of novel habitats could also vary similarly. Some demographic legacy effects could explain a counter-intuitive observation that a poorly dispersing old-forest lichen occupied a wider range of habitats in an area where it was rarer [106].

The scarcity of demographic evidence highlights a generally poor consideration of stochastic processes in the typically niche-based thinking of lichen ecologists. Yet dynamic populations that fluctuate along with the dispersal and local extirpation events, may become established in some habitats and remain absent from others due to a simple combination of stochastic events and environmental heterogeneity. For example, Neotropical assemblage-scale studies on epiphytes indicate that stochastic factors (notably dispersal) can be more important than environmental parameters there [234], or the latter might mostly matter at the microhabitat scale [235]. 

## 4. Evolutionary Processes Involved in Regional Habitat Use

### 4.1. Genetic and Phylogeographic Background

The first evolutionary inferences on habitat-specific lichen populations were obtained from the studies on chemotypes, which sometimes appeared habitat-specific [236]. Although, by current understanding, the correspondence of chemical races to phylogenetic lineages is highly variable [16,59], chemotypes are not likely to be selectively neutral [237]. Yet the mechanisms involved and their role for population divergence remain poorly studied. An experimental study found that lichenivorous gastropods favor a chemical race of *L. pulmonaria* that has fewer secondary substances; this race is less likely to occur at sites with a high abundance of lichenivores [238]. McCune et al. [239] reported that each chemotype of *Hypogymnia imshaugii* responded distinctly to climate in North America, as shown by the regression of occurrences of chemotypes against climatic variables. On the other hand, laboratory studies with axenically grown mycobionts confirm that their production of secondary compounds may much depend on the environmental conditions [240].

Perhaps the most convincing field evidence for intraspecific sorting of fungal genotypes comes from observations of habitat-specific lineages in sympatry. However, in the cases where this has been documented, the sympatric lineages probably evolved elsewhere—possibly in allopatry. In the relatively poorly dispersing *Lobaria pulmonaria* in Europe, there are two main lineages, one growing on beech and the other having a wider range of host trees [241]. These lineages appear to originate from different glacial refugia but, in a region of co-occurrence, they were nowadays segregated on the landscape. Similarly, in studies on *Xanthoria parietina* in Scandinavia, sympatric corticolous and saxicolous specimens represented partly differentiated lineages and, additionally, differentiation of an unusual Norwegian population inhabiting eutrophicated bark was observed. In Norway, such differentiation was estimated to date back at least 34 000 years, i.e., it had evolved elsewhere, and allopatric origin cannot be outruled [242,243]. Rock and bark substrates are known to differ in their nutrient status for *X. parietina* and, consequently, to affect its metabolism and the symbiotic relationship [244]. However, other lichens may respond to the corticolous-saxicolous substrate contrast with plasticity only (e.g., [245]). 

### 4.2. Evolutionary Consequences

Evolutionary consequences of the ecogeographic patterns described in Section 2 and Section 3 are not clear and mostly inferred from circumstantial evidence. Allopatric divergence of lichen-forming fungi might be most pronounced in climatically unfavorable regions where distinct local microhabitats frequently provide the necessary buffering (Section 3.2). In climates favorable for plant growth, lichens are often restricted to distinct competition-free microhabitats (Figure 4); such conditions support sympatric divergence, which can be fixed both by selection and genetic drift [33]. 

Yet a preliminary conclusion based on the available literature is that regionally habitat-adapted lineages of lichen-forming fungi are probably infrequent. The factors to consider are the phenotypic plasticity, effective gene flow over evolutionary time scales, slower adaptation, and slow genetic drift to remove ancestral haplotypes from typical lichen populations (e.g., [22,135,246]). Most likely, emergence of such lineages could involve particular population events or conditions, such as long-distance invasions or population bottlenecks [203]. Technically, their credible demonstration requires careful common-garden or translocation experiments, which are now emerging [247,248]. Even morpho-physiologically distinct ecotypes (found in many species; e.g., [249,250]) can be only slightly differentiated genetically (e.g., [111]). While Murtagh et al. [133] demonstrated that the fungal partner of *Xanthoria elegans* from extremely cold climates indeed retained enhanced growth rates over a wide temperature range, the ecosystem range addressed was enormous even for a lichen (from Antarctica to Alaska, and temperate North America to Europe). Furthermore, the symbiotic nature of the lichen has to be explicitly accounted for [251]. Thus, the fungal partners can adjust their metabolism by selectively incorporating photobiont lineages that are suitable in local thermal or chemical conditions (e.g., [138,246,252,253,254]; but see [255]). The photobionts, in turn, can possess unique inheritable mechanisms relevant for their symbiotic life-style and its physiological challenges [256], but study on the evolution of these characteristics has only begun [257].

Sometimes, regionally distinct habitat use and associated morphophysiological characters seem to be qualitatively similar to what is observed along sharp local, but continuous, gradients. For example, Harris [258] highlighted intraspecific morphophysiological differences of epiphytes from tree tops to bottoms. Rikkinen [259] described profound habitat and morphological differences in *Pseudevernia furfuracea* between two sites only 150 m apart in Finland, where “the difference in their effective temperature sums was equivalent to the macroclimatic difference generally associated with a distance interval of almost 800 km in a north-south direction”. Similar kind of short-distance variation along steep environmental gradients has been found in the genetic structure [260] and secondary chemistry of lichen-forming fungi [261,262]. Thus, selection pressures across regions may not always qualitatively differ from smaller scale variation within ecosystems or along topographic gradients in a landscape.

These lines of evidence point to that, in terms of habitat-relationships, plasticity may be a superior strategy over local specialization for lichens in most cases. Consequently, a major evolutionary process may be the evolution of plasticity to exploit variable resources available and to switch to novel habitats. Lichens inhabiting non-native natural substrates would deserve study in this respect. Another general trait expected to be under selective pressure is the dispersal ability. It probably pays off not to be too selective when substrate diversity is very high and the colonization of emerging free surfaces by competitors is rapid. Similar thinking might also be used for relating lichens’ ability of long-distance dispersal to plasticity, but there are alternatives: either dispersal ability might be selected for because of a very specific and rare habitat (e.g., [263]), or it induces plasticity (i.e., the use of wider conditions which are more likely to be encountered at longer distances).

## 5. Conservation Implications

For lichen conservation, habitat conservation and management are often the sole practical options [30,264]. Spatial variation in habitat relationships thus deserves attention for sustaining lichen diversity, notably for sparsely distributed species. We outline four fields of conservation actions that are supported by the current knowledge. 

I. *Assessing regional threats on ecosystem integrity.* For conserving lichen-forming species, Red Listing is a well standardized, but data-demanding, regional approach (e.g., [122,265]). However, since adding spatial resolution to that procedure would further increase its data requirements, complementary approaches are needed for regional habitat patterns. We found that most within-species variation in lichen habitat relationships revealed plastic adaptation to available combinations of ecological factors, rather than evolutionary lineages. This highlights regional ecosystem integrity, resilience, and related risk assessments (long-term trends; disasters; novel threats) as a major approach for lichen conservation (e.g., [266,267,268]), for which sensitive species might serve as indicators [269].

We exemplify this point by considering the dieback of the European ash (*Fraxinus excelsior*). The dieback, caused by the invasive exotic ascomycete *Hymenoscyphus fraxineus*, has roughly halved the ash populations in the continent in two decades, and continues to expand [270,271]. Multiple assessments indicate that this process can threaten the specific epiphyte assemblages of ash (e.g., [272,273,274,275]). From 548 ash-inhabiting lichens in the United Kingdom, thirteen have been recently listed as potentially threatened by the dieback and 49 species as prone to decline [274]. However, when we compiled similar lists for four North-Europe countries (Table S3), we found that only 94 species from a total of 343 lichens are consistently ash-inhabiting. Moreover, while 38 species are known on ash in a single country and inhabit other substrates elsewhere, some regional occurrences include threatened populations (e.g., *Sclerophora coniophaea* in Sweden, *Lopadium disciforme* in Estonia; *Chaenotheca hispidula* in Lithuania; *Cliostomum corrugatum* in northeastern Poland). In fact, no species listed as threatened by Mitchell et al. [274] has a similar combination of host-specificity and risk level throughout Northern Europe (Table S3). These findings imply that (i) most species will be affected by the dieback only regionally or even locally [276], which blurs the link between regional extinction risk and management priorities (see [277]); however, (ii) the ecosystem-scale threat from the dieback to epiphytes is universal and can be addressed for its ecological significance. Species resolution can then be added for local interventions (e.g., transplanting) to save threatened endemics.

II. *Maintaining long-term structural heterogeneity of ecosystems.* Our review indicated that (i) regional microhabitat shifts within and across habitat types are common in lichens, but (ii) only some consistent patterns (notably in relation to moisture and the chemical environments) are well documented (Section 3.2), and (iii) the genetic, plasticity-related, and demographic mechanisms of those shifts remain poorly understood (e.g., [129]). Furthermore, (iv) the ecosystem structures are changing, sometimes to novel (unprecedented) forms (e.g., [278]).

For example, dead branches in tree canopies appear marginal microhabitats for lignicolous lichens in Fennoscandian managed forests [279]. Yet this finding cannot be directly adopted in the management of temperate forests where, perhaps due to competition by plants and bryophytes, many lichens appear increasingly deadwood-dependent and competitively excluded from near-ground microhabitats (Table 1; Section 3.2.3). The management of dead wood for lichens thus requires regional approaches.

In brief, there are only limited abilities to explicitly plan for future buffering or new habitat-provision functions of most lichen microhabitats. Identifying new substrates, such as host trees for epiphytes, might be an exception [280,281]. However, one can manage for stable microenvironment diversity in any ecosystem, which might provide habitat for lichens under changing broad-scale conditions (e.g., [157]). While some approaches to that are well elaborated (e.g., a necessity to maintain ancient trees and sufficient areas of structurally heterogeneous woodland), others are not—for example, in urban, industrial, or agricultural areas, or in the ecosystems invaded by exotic species. A broad ecosystem heterogeneity framework to lichen conservation would realize the precautionary principle of the environmental management, which remains an underdeveloped tool in biodiversity conservation. 

III. *Considering lichen ecotypes as conservation targets.* A precautionary approach at the species level would be to maintain a documented ecological variation within lichen populations, even if its adaptive significance is not clear. It is nevertheless likely that different ecologies expose lichen partners and their relationships to potentially significant selection pressures. Unless proven otherwise, ‘ecotypes’ could thus be seen as potential evolutionarily significant units or a bet-hedging strategy for addressing the climate change and land-use change-related challenges to biodiversity. This option (and the habitat-shift potential more generally) seems to be so far absent in the mainstream thinking on climate change response in lichens (e.g., [282]).

IV. *Reconsidering broad-scale biodiversity indicators.* Despite many problems [283], there are few alternatives to biological indicators when it comes to assessing the biodiversity significance of environmental change, particularly of anthropogenic and novel pressures. Broad-scale assessments (e.g., comparing the performance of countries) are particularly useful for informing international and national environmental policies. To represent lichen diversity, using sets of widely distributed species or well-known guilds for such comparisons appears to logically follow (e.g., [107,269,284,285]). However, using such lists would require conceptual clarification of how regional habitat requirements and habitat buffering are accounted for. For example, varying niche breadths along climatic gradients (Section 3.3) may reveal varying impacts of similar levels of land-use intensity, which may or may not be appropriate as a basis for comparisons of environmental performance.

## 6. Conclusions

- It has been long observed that some lichen-forming fungi inhabit regionally distinct habitats, which cannot be explained by available habitat types or substrates alone. However, no theoretical framework has been developed to capture this phenomenon despite its apparent links to the basic lichen biology and to biodiversity conservation. We organized these observations around habitat shifts at two scales (macro- and microhabitat), their likely causal mechanisms, and possible evolutionary consequences.
- We report that consistent intraspecific habitat patterns can be usually explained with regional physiological challenges (including physiological trade-offs) or, in favorable environments, coping with competition or predation. Replicated evidence exists for three patterns: (a) regional limiting factors excluding a species from a part of its microhabitat range in suboptimal areas; (b) microhabitat shifts buffering regionally adverse macroclimates; (c) substrate suitability changed by the chemical environment, notably air pollution. There is also a role for switching algal partners in different regions and habitats, but no consistent patterns emerged based on the current evidence.
- The processes creating and maintaining regional lichen-habitat relationships are generally known (adaptive and plasticity-related responses; demographic processes and events), but not explicitly described. Thus, lichen habitat responses in the future cannot be predicted, particularly given the likely ecosystem changes toward unprecedented states due to anthropogenic pressures. For example, regional microrefugia to buffer changing climate appear to be often assumed, but the actual evidence is weak.
- To deal with the uncertainty, effective lichen conservation might integrate a precautionary approach to ecosystem conservation, restoration, and management. There are good reasons of lichens becoming a part of ecosystem heterogeneity, integrity, and resilience considerations.

## Figures and Tables

**Table 1:** Examples of geographically varying habitat preferences of lichens in the cases where different habitat availability has been accounted for or can be assumed. See [50,51] for a collection of earlier observations, notably on shifts in substrate use.

Habitat Relationship	Geographic Scale Studied	Evidence [Source] ^a^	Mechanism ^b^
Phorophyte specificity in epiphytes	North America vs. Europe	For cyanolichens, conifer hosts more frequent than hardwoods in N America; opposite in Europe, despite many species shared [95]	2 *
	Britain (BR) vs. subcontinental Europe (SC)	Varying tree-species specificity among lichens of conservation concern: On old *Quercus robur*, only 7 specialists (*Bactrospora dryina*, *Calicium adspersum*, *C. quercinum*, *Caloplaca lucifuga*, *Lopadium disciforme, Peltigera horizontalis*, and *Pertusaria flavida*) shared in the two regions; the rest on the BR list absent or not confined to oak in SC, where six species mainly found on oak not listed as “faithful” to oak in BR (Table S1). *Betula* spp. harbors more species in BR, including those typical of other hardwoods in SC (Table S2).	3
	North Europe	Lichens found on European ash in Sweden, Estonia, Lithuania, NE Poland: 94 of 343 species in all countries, 38 in one country; spatial pattern in adjacent countries in 14 species (Table S3).	3
	Fennoscandia (FS) vs. Estonia (EE)	Regional preferences for Scots pine vs. Norway spruce: *Cladonia ochrochlora* and *Lepraria jackii* prefer spruce in FS, but pine in EE; *Ochrolechia androgyna* and *Pseudevernia furfuracea* prefer spruce and *Parmeliopsis hyperopta* pine in EE, indifferent in FS [96].	unclear
	Climatic gradients in Norway	Regional substrate use in epiphytic crustose lichens; e.g., *Micarea coppinsii* on tree bark in addition to acidic shrubs in optimal conditions; some species vary in shade tolerance and phorophytes [97].	2–4
Dead-wood specificity	Pacific Northwest (PNW) vs. Fennoscandia (FS)	Of 65 crustose lichens present in both regions and obligately lignicolous in at least one, 4 species not known on wood in the other region: *Absconditella celata*, *A. trivialis* and *Micarea alabastrites* strictly wood-inhabiting in PNW but terricolous, bryicolous or corticolous in FS; *Caloplaca furfuracea* confined to anthropogenic wood in FS and to bark in PNW. Many species obligate lignicoles in one region and facultative in the other [98].	1–4
	North-south gradient from Fennoscandia to Lithuania	Twenty-six species inhabit wood facultatively or not at all in Fennoscandia, but become obligate or facultative lignicoles further south (e.g., *Chaenotheca chlorella*, *Cladonia floerkeana*, *Icmadophila ericetorum*) [A].	1–2
Rock type preference	global	16% of 75 serpentine specialists of Europe and America elsewhere in Middle Urals (7 on granite; 5 on limestone); serpentine (113 species) and granite biota (70) still distinct (14 species shared) [99].	1–3
Shift between corticolous and saxicolous substrate use	global	Many observations worldwide [51]. Attributed to usable water source (dew) in *Menegazzia terebrata*, which is epiphytic in oceanic regions but uses exposed rocks in dry regions of Norway [100].	4
	global	Three nitrophytic macrolichens have latitudinal substrate shifts; *Physcia caesia* also 20th century switch to eutrophicated tree bark in the Netherlands [101]	2
	Central Europe vs. elsewhere	Freshwater saxicolous lichens frequent on tree roots in the Alps and NE Europe but rarely in Central European lowlands, perhaps due to eutrophication and silting of water bodies) [102]. Air pollution may also explain why some seriously declined corticolous lichens in Czechia retain remnant populations on rocks [103].	2 *
Forest type specificity	Baltic countries	Among 30 species in two contrasting forest types in Estonia (dry pine and eutrophic mixed forests), *Cladonia cenotea* and *Pertusaria amara* inhabit a single type in Lithuania. Attributed to ground-level competition and light conditions on tree trunks, respectively [A].	2
	Finland	Moisture demanding species concentrate to spruce swamps more in southern than in middle-boreal sites [69].	2
Old-growth affinity	Climatic gradients in the Pacific Northwest	Most old-growth-dependent epiphytic macrolichens of inland areas use wider successional stages in oceanic areas [104,105]. Some continental macrolichens exhibit an inverse relationship, being restricted to old-forest canopies in oceanic areas [106].	2–3
	Sweden	Several species only regionally old-growth specific, e.g., *Arthonia spadicea*, *A. vinosa*, *Bacidia rubella*, *Chaenotheca brachypoda* [107]	unclear
Use of artificial substrates	The Netherlands	Stone churches host regionally distinct lichen assemblages [37].	2
	N America vs. N Europe	*Cladonia parasitica* common on exposed fence rails in N America, not found on worked timber in Europe [108,109]. See also Figure 2.	unclear

^a^ sources: [A] P. Lõhmus and J. Motiejūnaitė, unpublished data. ^b^ likely explanations as on Figure 3 (* explanation of the original study).

## Data Availability

The original data used in this paper has been presented in the Supplementary Materials.

## Supplementary Materials

The following supporting information can be downloaded at: https://www.mdpi.com/article/10.3390/jof9030341/s1, Table S1: Consistency in oak preference in epiphytic lichens of conservation concern in Britain, Lithuania and NE Poland, Table S2: Consistency in occupancy of birch (*Betula* sp.) by epiphytic lichens of conservation concern in Britain, Lithuania, Estonia and NE Poland, Table S3: Occupancy of European ash (*Fraxinus excelsior*) by epiphytic lichens in Northern Europe.
