# Supplementary material for: Regionally Varying Habitat Relationships in Lichens: The Concept and Evidence with an Emphasis on North-Temperate Ecosystems
Source: J Fungi (Basel). 2023 Mar 10;9(3):341. doi: 10.3390/jof9030341 (PMC10056719; doi:10.3390/jof9030341)
Supplement: Supplementary file 1 [file jof-09-00341-s001.zip › jof-2140819-supplementary.pdf]

A. Lõhmus, J. Motiejunaite & P. Lõhmus
Supplementary material. Use by epiphytic lichens of three species of host-trees across Europe.

Table S1
Consistency in oak preference (++ grows mainly on oak; + grows on oak; – does not grow on oak) in epiphytic lichens of conservation concern in Britain, Lithuania and NE Poland (see list of sources below of the table). Included are the species, which are known from all three countries and, in at least one country, grow mainly on oak and are rare or threatened. The seven species consistently preferring oak are indicated in **Bold.**

Species	Britain	Lithuania	NE Poland
Arthonia arthonioides	+	++	++
Arthonia didyma	++	+	+
Arthonia vinosa	+	++	++
Bactrospora dryina	++	++	++
Calicium adspersum	++	++	++
Calicium quercinum	++	++	++
Calicium viride	+	++	++
Caloplaca lucifuga	++	++	++
Catinaria atropurpurea	++	–	+
Chaenotheca phaeocephala	+	++	++
Chrysothrix candelaris	+	++	++
Cliostomum corrugatum	–	++	++
Hypotrachyna revoluta	++	–	+
Leptogium teretiusculum	++	–	–
Lobaria pulmonaria	++	+	+
Lopadium disciforme	++	++	++
Nephroma parile	++	–	–
Peltigera horizontalis	++	++	++
Pertusaria flavida	++	++	++
Thelotrema lepadinum	++	–	+

Sources:
Britain:
Rose, F. The epiphytes of oak. In *The british oak, its history and natural history* (eds. MG Morris; FH Perring). 1974 pp 250–273.
Smith, C.W.; Aptroot, A.; Coppins, B.J.; Fletcher, A.; Gilbert, O.L.; James, P.W.; Wolseley, P.A. (eds). 2009. *The lichens of Great Britain and Ireland*. Second edition. London: British Lichen Society
Lithuania: Motiejunaite, J. unpubl. data
NE-Poland:
Cieśliński, S. Distribution atlas of lichens (Lichenes) in North-Eastern Poland. 2003. *Phytocoenosis* 15, 430.
Zalewska, A. Ecology of lichens of the Puszcza Borecka Forest (NE Poland). *W. Szafer Institute of Botany, Polish Academy of Sciences*. 2012.

Table S2
Consistency in occupancy of birch (*Betula* sp.) by epiphytic lichens of conservation concern in Britain, Lithuania, Estonia and NE Poland (see list of sources below of the table). Included are the species, which are known from all four countries and, in at least one country, inhabit birch and are rare or threatened. (+ grows on birch, – does not grow on birch, * only historical occurrence on birch).

Species	Britain	Lithuania	Estonia	NE Poland
Acrocordia gemmata	–	+	–	–
Anisomeridium polypori	+	–	+	–
Arthonia didyma	+	–	–	–
Arthonia leucopellaea	+	–	+	–
Arthonia vinosa	+	–	+	–
Bacidia arceutina	+	–	+	–
Cetrelia olivetorum (s. l.)	+	–	–	+
Chaenotheca furfuracea	+	–	+	+
Chrysothrix candelaris	+	–	–	+
Cyphelium inquinans	+	–	–	+
Sclerophora nivea	+	–	–	–
Sclerophora peronella	+	–	–	–
Fellhaneropsis vezdae	+	–	–	–
Lecanactis abietina	+	–	+	+
Lobaria pulmonaria	+	–	–	*
Menegazzia terebrata	+	–	+	+
Nephroma parile	+	–	–	–
Psilolechia lucida	+	–	+	+
Thelotrema lepadinum	+	–	+	–

Sources:
Britain:
Coppins, B.J. Epiphytes of birch. *Proc. R. Soc. of Edinburgh*. 1984 *Section* B *85*, 115–128.
Estonia:
Lõhmus, P.; Lõhmus, A. The potential of production forests for sustaining lichen diversity: a perspective on sustainable forest management. *Forests*. 2019 *10*, 1063. (raw data)
Lithuania: Motiejunaite, J. unpubl. data
NE-Poland:
Cieśliński, S. Distribution atlas of lichens (Lichenes) in North-Eastern Poland. 2003. Phytocoenosis 15, 430.
Zalewska, A. Ecology of lichens of the Puszcza Borecka Forest (NE Poland). *W. Szafer Institute of Botany, Polish Academy of Sciences*. 2012.

Table S3. Occupancy of European ash (*Fraxinus excelsior*) by epiphytic lichens in Northern Europe. Abbreviations: n.a. - not known from the country, – present in the country, but not found on ash, + grows on ash, not threatened, ++ a rare and/or threatened species growing on ash. Blue highlights occupancy on the ach in one country only. List of sources see below of the table.

Species	Sweden	Estonia	Lithuania	NE Poland
Acrocordia cavata	+	+	++	-
Acrocordia gemmata	+	+	+	+
Agonimia allobata	+	-	++	-
Agonimia tristicula	+	-	n.a.	-
Alyxoria varia	+	+	+	+
Amandinea punctata	+	+	+	+
Anaptychia ciliaris	+	+	+	+
Anisomeridium biforme	+	++	n.a.	+
Anisomeridium polypori	+	++	+	-
Arthonia arthonioides	-	-	++	++
Arthonia byssacea	++	++	+	++
Arthonia cinnabarina	++	-	n.a.	-
Arthonia didyma	+	++	-	++
Arthonia dispersa	+	-	+	+
Arthonia leucopellaea	+	+	-	++
Arthonia mediella	+	+	-	+
Arthonia muscigena	+	-	-	-
Arthonia punctiformis	+	++	+	n.a.
Arthonia radiata	+	+	+	+
Arthonia spadicea	+	+	+	+
Arthonia vinosa	+	+	++	++
Arthopyrenia ranunculospora	-	n.a.	n.a.	+
Arthothelium ruanum	+	+	+	+
Arthothelium spectabile	n.a.	-	n.a.	+
Arthrosporum populorum	-	++	-	-
Bacidia absistens	++	n.a.	n.a.	n.a.
Bacidia arceutina	+	+	+	++
Bacidia beckhausii	+	+	++	++
Bacidia biatorina	-	++	-	-
Bacidia brandii	n.a.	n.a.	+	n.a.
Bacidia circumspecta	+	-	-	n.a.
Bacidia fraxinea	+	+	++	++
Bacidia hemipolia	+	-	n.a.	n.a.
Bacidia incompta	-	++	-	++
Bacidia laurocerasi	-	++	+	-
Bacidia polychroa	+	+	+	++
Bacidia rosella	+	n.a.	++	++
Bacidia rubella	+	+	+	+
Bacidia subincompta	+	+	+	++
Bacidina assulata	n.a.	n.a.	++	++
Bacidina caligans	+	-	n.a.	n.a.
Bacidina chloroticula	+	-	-	-

Bacidina inundata	-	-	+	-
Bacidina phacodes	-	-	n.a.	+
Bacidina sulphurella	+	-	+	+
Bactrospora corticola	+	n.a.	n.a.	n.a.
Biatora albohyalina	+	++	++	-
Biatora chrysantha	-	++	-	+
Biatora efflorescens	-	+	-	-
Biatora epixanthoides	+	+	++	n.a.
Biatora helvola	+	+	++	n.a.
Biatora meiocarpa	n.a.	n.a.	++	n.a.
Biatora ocelliformis	+	+	++	+
Biatora sphaeroidiza	+	-	n.a.	n.a.
Biatoridium monasteriense	+	++	++	++
Bilimbia sabuletorum	+	+	+	-
Bryoria capillaris	-	+	-	-
Bryoria fuscescens	-	+	-	+
Bryoria implexa	-	+	-	+
Buellia disciformis	+	+	-	++
Buellia erubescens	-	+	-	++
Buellia griseovirens	+	+	+	+
Buellia schaereri	-	+	+	-
Calicium quercinum	-	+	-	-
Calicium salicinum	+	+	+	+
Calicium viride	-	-	+	+
Caloplaca cerina	+	+	-	+
Caloplaca cerinella	+	-	-	-
Caloplaca chlorina	+	-	n.a.	-
Caloplaca chrysophthalma	+	+	n.a.	n.a.
Caloplaca citrina agg.	+	-	-	-
Caloplaca ferruginea	+	-	n.a.	n.a.
Caloplaca flavorubescens	+	+	+	++
Caloplaca herbidella	+	-	-	n.a.
Caloplaca lucifuga	-	++	-	-
Caloplaca luteoalba	++	n.a.	n.a.	n.a.
Caloplaca obscurella	+	-	++	-
Caloplaca pyracea	+	-	+	+
Caloplaca subathallina	+	n.a.	n.a.	n.a.
Caloplaca ulcerosa	++	-	n.a.	n.a.
Candelaria pacifica	-	+	-	+
Candelariella efflorescens	+	n.a.	++	+
Candelariella reflexa	++	-	+	+
Candelariella vitellina	-	+	+	+
Candelariella xanthostigma	+	+	+	+
Catillaria croatica	n.a.	n.a.	+	n.a.
Catillaria nigroclavata	+	+	+	+
Catinaria atropurpurea	+	++	-	++
Cetrelia olivetorum s.lat.	-	++	++	++
Chaenotheca brachypoda	-	+	+	++
Chaenotheca chlorella	-	-	++	++
Chaenotheca chrysocephala	-	+	-	+

Chaenotheca cinerea	++	++	++	n.a.
Chaenotheca furfuracea	-	+	+	++
Chaenotheca gracilenta	-	+	++	++
Chaenotheca hispidula	-	-	++	-
Chaenotheca phaeocephala	-	+	+	-
Chaenotheca stemonea	-	+	-	-
Chaenotheca subroscida	-	+	n.a.	n.a.
Chaenotheca trichialis	+	+	+	+
Chaenotheca xyloxena	-	+	+	-
Chrysothrix candelaris	+	+	+	+
Cladonia caespiticia	-	-	-	++
Cladonia cenotea	-	+	-	-
Cladonia chlorophaea	+	+	+	+
Cladonia coniocraea	+	+	+	+
Cladonia digitata	-	+	-	-
Cladonia fimbriata	+	+	+	+
Cladonia ochrochlora	-	+	-	+
Cladonia ramulosa	-	-	+	-
Cladonia squamosa	-	-	+	-
Cliostomum corrugatum	-	-	-	++
Cliostomum flavidulum	+	-	n.a.	n.a.
Cliostomum griffithii	+	+	-	-
Coenogonium luteum	++	++	n.a.	-
Coenogonium pineti	+	+	+	+
Collema flaccidum	+	++	++	-
Collema nigrescens	++	-	-	n.a.
Collema occulltatum	++	-	n.a.	++
Collema subflaccidum	++	n.a.	n.a.	n.a.
Cyphelium sessile	++	-	n.a.	n.a.
Degelia plumbea	++	n.a.	n.a.	n.a.
Diploicia canescens	++	n.a.	n.a.	n.a.
Diplotomma alboatrum	+	-	++	-
Diplotomma pharcidium	+	+	++	-
Enterographa crassa	++	n.a.	n.a.	n.a.
Eopyrenula leucoplaca	+	++	n.a.	n.a.
Evernia prunastri	+	+	+	+
Fellhanera gyrophorica	n.a.	++	+	++
Fellhaneropsis vezdae	-	-	++	-
Flavoparmelia caperata	++	-	-	++
Fuscidea arboricola	+	+	-	-
Fuscidea pusilla	-	+	-	n.a.
Fuscopannaria mediterranea	+	n.a.	n.a.	n.a.
Graphis scripta	+	+	+	+
Gyalecta derivata	-	n.a.	++	n.a.
Gyalecta flotowii	++	-	++	++
Gyalecta truncigena	++	+	++	++
Gyalecta ulmi	++	++	++	++
Hypocenomyce scalaris	+	-	-	-
Hypogymnia farinacea	+	-	-	++
Hypogymnia physodes	+	+	+	+

Hypogymnia tubulosa	+	+	+	+
Hypotrachyna revoluta	++	n.a.	++	++
Imshaugia aleurites	+	+	-	-
Lecanactis abietina	-	+	-	-
Lecania cyrtella	+	+	+	+
Lecania cyrtellina	+	-	-	++
Lecania dubitans	+	-	n.a.	n.a.
Lecania fuscella	+	++	-	++
Lecania hyalina	+	+	+	+
Lecania koerberiana	+	-	n.a.	-
Lecania naegelii	+	+	+	++
Lecania prasinoides	n.a.	n.a.	+	-
Lecanora albella	-	+	-	-
Lecanora albellula	+	-	-	-
Lecanora allophana	+	+	+	+
Lecanora argentata	+	+	+	+
Lecanora carpinea	+	+	+	+
Lecanora chlarotera	+	+	+	+
Lecanora compallens	n.a.	++	-	n.a.
Lecanora conizaeoides	-	+	-	+
Lecanora expallens	+	+	+	+
Lecanora glabrata	+	-	n.a.	+
Lecanora hagenii	+	+	+	+
Lecanora impudens	+	-	n.a.	n.a.
Lecanora intumescens	-	++	-	++
Lecanora leptyrodes	-	+	n.a.	+
Lecanora populicola	-	+	-	-
Lecanora pulicaris	+	+	+	+
Lecanora rugosella	-	+	-	+
Lecanora saligna	-	+	-	+
Lecanora sambuci	+	+	++	-
Lecanora symmicta	+	+	+	+
Lecanora thysanophora	n.a.	++	+	+
Lecanora umbrina	-	n.a.	n.a.	+
Lecanora varia	+	+	+	+
Lecanora viridissima	+	n.a.	n.a.	n.a.
Lecidea erythrophaea	+	++	++	++
Lecidea nylanderi	+	+	-	-
Lecidella achristotera	+	n.a.	n.a.	n.a.
Lecidella elaeochroma	+	+	+	+
Lecidella euphorea	+	+	-	+
Lecidella flavosorediata	+	+	++	++
Lecidella scabra	+	-	n.a.	n.a.
Lecidella subviridis	-	+	-	n.a.
Lepraria crassissima	-	++	n.a.	n.a.
Lepraria eburnea	+	+	-	-
Lepraria elobata	+	+	+	-
Lepraria incana	+	+	-	+
Lepraria jackii	-	+	-	-
Lepraria lobificans	+	+	+	+

Lepraria rigidula	-	n.a.	-	+
Lepraria vouauxii	-	-	+	+
Leptogium cyanescens	+	-	n.a.	n.a.
Leptogium intermedium	+	-	n.a.	n.a.
Leptogium lichenoides	+	+	++	n.a.
Leptogium saturninum	-	++	-	++
Leptogium teretiusculum	+	++	-	-
Lobaria amplissima	++	n.a.	n.a.	n.a.
Lobaria pulmonaria	++	++	++	++
Lopadium disciforme	-	++	-	-
Loxospora elatina	-	+	-	+
Megalaria grossa	++	++	n.a.	n.a.
Megalaria laureri	+	n.a.	n.a.	-
Megalaria pulverea	+	n.a.	n.a.	n.a.
Melanelixia fuliginosa	+	+	+	+
Melanelixia subargentifera	+	+	+	+
Melanelixia subaurifera	+	+	+	++
Melanohalea exasperata	+	+	-	++
Melanohalea exasperatula	+	+	+	+
Melanohalea olivacea	-	+	-	-
Melanohalea septentrionalis	-	++	-	n.a.
Melanohalea elegantula	+	-	n.a.	+
Melanohalea laciniatula	+	n.a.	n.a.	n.a.
Menegazzia terebrata	++	++	++	++
Micarea nitschkeana	+	-	-	++
Micarea peliocarpa	-	+	++	-
Micarea prasina	+	+	+	+
Mycobilimbia carneoalbida	+	+	n.a.	-
Mycobilimbia epixanthoides	+	-	-	n.a.
Mycobilimbia hypnorum	+	-	n.a.	-
Mycoporum antecellens	+	-	n.a.	n.a.
Nephroma laevigatum	-	++	n.a.	-
Nephroma parile	-	++	-	-
Normandina acroglypta	+	++	+	n.a.
Ochrolechia androgyna s.lat	+	+	-	+
Ochrolechia arborea	+	+	++	++
Ochrolechia microsticoides	-	+	-	+
Ochrolechia pallescens	-	++	-	++
Ochrolechia sordidogrisea	+	n.a.	n.a.	n.a.
Ochrolechia szatalaensis	-	++	n.a.	n.a.
Ochrolechia subviridis	+	-	n.a.	n.a.
Ochrolechia turneri	+	++	+	+
Opegrapha atra	+	++	-	+
Opegrapha culmigena	+	-	n.a.	n.a.
Opegrapha niveoatra	+	-	+	+
Opegrapha ochrocheila	+	++	n.a.	n.a.
Opegrapha rufescens	+	+	+	+
Opegrapha sorediifera	+	++	-	n.a.
Opegrapha vermicellifera	+	n.a.	-	++
Opegrapha vulgata	+	+	++	-

Pachyphiale fagicola	+	++	++	++
Pannaria conoplea	++	n.a.	n.a.	n.a.
Parmelia ernstiae	-	-	+	n.a.
Parmelia saxatilis	+	+	+	+
Parmelia submontana	+	-	+	++
Parmelia sulcata	+	+	+	+
Parmeliella triptophylla	+	++	-	n.a.
Parmelina tiliacea	+	++	+	+
Parmeliopsis ambigua	+	+	-	+
Peltigera canina	+	-	-	-
Peltigera didactyla	-	+	-	-
Peltigera polydactylon	-	+	+	+
Peltigera praetextata	+	+	+	+
Pertusaria albescens	+	+	+	+
Pertusaria amara	+	+	+	+
Pertusaria coccodes	+	+	+	+
Pertusaria coronata	+	+	-	++
Pertusaria flavida	+	++	+	+
Pertusaria hemisphaerica	+	+	-	+
Pertusaria hymenea	+	n.a.	n.a.	++
Pertusaria leioplaca	+	+	-	+
Pertusaria multipuncta	+	-	n.a.	n.a.
Pertusaria ophthalmiza	+	-	-	+
Pertusaria pertusa	+	+	+	+
Pertusaria pupillaris	+	-	+	+
Phaeophyscia ciliata	-	+	-	-
Phaeophyscia endophoenicea	+	-	-	-
Phaeophyscia nigricans	-	+	+	+
Phaeophyscia orbicularis	+	+	+	+
Phlyctis agelaea	+	+	+	++
Phlyctis argena	+	+	+	+
Physcia adscendens	+	+	+	+
Physcia aipolia	+	+	+	+
Physcia caesia	-	+	-	+
Physcia dubia	+	+	+	+
Physcia leptalea	-	+	n.a.	n.a.
Physcia stellaris	+	+	+	+
Physcia tenella	+	+	+	+
Physconia detersa	-	++	-	-
Physconia distorta	+	+	+	+
Physconia enteroxantha	+	+	+	+
Physconia grisea	-	++	+	+
Physconia perisidiosa	+	+	+	+
Placynthiella dasaea	-	-	-	++
Placynthiella icmalea	-	+	-	+
Platismatia glauca	-	+	+	+
Pleurosticta acetabulum	+	+	+	+
Polycauliona candelaria	-	+	+	+
Polycauliona phlogina	n.a.	n.a.	++	n.a.
Polycauliona polycarpa	-	+	+	+

Pseudevernia furfuracea	+	+	+	+
Pseudosagedia aenea	+	++	-	+
Psilolechia clavulifera	-	+	-	-
Pyrenula laevigata	-	++	++	++
Pyrenula nitida	-	-	++	+
Pyrenula nitidella	++	++	+	++
Pyrrhospora quernea	++	+	-	-
Ramalina baltica	-	+	+	+
Ramalina calicaris	+	-	n.a.	-
Ramalina elegans	+	-	n.a.	n.a.
Ramalina farinacea	+	+	+	+
Ramalina fastigiata	+	+	+	+
Ramalina fraxinea	+	+	+	+
Ramalina pollinaria	+	+	-	+
Reichlingia leopoldii	n.a.	++	+	+
Rinodina efflorescens	+	++	+	n.a.
Rinodina exigua	+	+	-	+
Rinodina laevigata	+	n.a.	n.a.	n.a.
Rinodina polyspora	+	n.a.	n.a.	n.a.
Rinodina pyrina	+	+	-	+
Rinodina sophodes	+	-	+	n.a.
Ropalospora viridis	+	+	+	+
Schismatomma decolorans	-	n.a.	n.a.	++
Schismatomma pericleum	+	-	++	-
Sclerophora amabilis	++	n.a.	n.a.	n.a.
Sclerophora coniophaea	++	-	-	-
Sclerophora farinacea	++	++	++	n.a.
Sclerophora pallida	+	+	+	++
Sclerophora peronella	-	+	-	++
Scoliciosporum chlorococcum	+	+	+	+
Scoliciosporum sarothamni	+	+	+	n.a.
Strangospora deplanata	+	n.a.	++	-
Strangospora pinicola	-	-	++	+
Strigula stigmatella	-	-	++	n.a.
Tephromela atra	+	+	++	-
Thelenella pertusariella	+	n.a.	-	n.a.
Thelopsis rubella	+	n.a.	n.a.	n.a.
Thelotrema lepadinum	-	++	++	++
Trapeliopsis flexuosa	-	+	-	-
Tuckermannopsis chlorophylla	+	+	-	+
Usnea filipendula	-	+	-	+
Usnea hirta	+	+	-	+
Usnea subfloridana	+	+	-	+
Vezdaea aestivalis	-	++	-	n.a.
Violella fucata	+	+	+	+
Vulpicida pinastri	+	-	-	+
Xanthomendoza fulva	+	+	++	++
Xanthoria parietina	+	+	+	+
Zwackhia viridis	+	++	++	+

Sources:

Sweden

Hallingbäck, T. *Ekologisk katalog över lavar*. Artdatabanken i samarbete med Naturvårdsverket. 1995.

Johansson, P.; Rydin H.; Thor, G. Tree age relationships with epiphytic lichen diversity and lichen life history traits on ash in southern Sweden. *Ecoscience*. 2007 *14*, 81–91.

Jönsson, M.T.; Thor, G. Estimating coextinction risks from epidemic tree death: affiliate lichen communities among diseased host tree populations of Fraxinus excelsior.

Estonia:

Jüriado, I.; Liira, J.; Paal, J.; Suija, A. Tree and stand level variables influencing diversity of lichens on temperate broad-leaved trees in boreo-nemoral floodplain forests. *Biodiver. Conserv.* 2009 *18*, 105–125.

Jüriado, I.; Liira, J.; Paal, J. Diversity of epiphytic lichens in boreo-nemoral forests on the North-Estonian limestone escarpment: the effect of tree level factors and local environmental conditions. *Lichenologist*. 2009 *41*, 81–94.

Leppik, E.; Jüriado, I.; Liira, J. Changes in stand structure due to the cessation of traditional land use in wooded meadows impoverish epiphytic lichen communities. *Lichenologist*. 2011 *43*, 257–74.

Lõhmus, P.; Lõhmus, A. The potential of production forests for sustaining lichen diversity: a perspective on sustainable forest management. *Forests*. 2019 *10*, 1063. (raw data)

Lithuania: Motiejunaite, J. unpubl. data

NE-Poland:

Cieśliński, S. Distribution atlas of lichens (Lichenes) in North-Eastern Poland. 2003. Phytocoenosis 15, 430.

Zalewska, A. Ecology of lichens of the Puszcza Borecka Forest (NE Poland). *W. Szafer Institute of Botany, Polish Academy of Sciences*. 2012.
